# Cryptococcal Meningitis and Post-Infectious Inflammatory Response Syndrome in a Patient With X-Linked Hyper IgM Syndrome: A Case Report and Review of the Literature

**DOI:** 10.3389/fimmu.2021.708837

**Published:** 2021-07-15

**Authors:** Lorenza Romani, Peter Richard Williamson, Silvia Di Cesare, Gigliola Di Matteo, Maia De Luca, Rita Carsetti, Lorenzo Figà-Talamanca, Caterina Cancrini, Paolo Rossi, Andrea Finocchi

**Affiliations:** ^1^ Unit of Immune and Infectious Diseases, Bambino Gesu’ Children’s Hospital, IRCCS, Rome, Italy; ^2^ Laboratory of Clinical Immunology and Microbiology, National Institutes of Health, Bethesda, MD, United States; ^3^ Department of Systems Medicine, University of Rome Tor Vergata, Rome, Italy; ^4^ B Cell Physiopathology Unit, Immunology Research Area, Bambino Gesu’ Children’s Hospital, IRCCS, Rome, Italy; ^5^ Neuroradiology Unit, Imaging Department, Bambino Gesu’ Children’s Hospital, IRCCS, Rome, Italy

**Keywords:** X-linked Hyper IgM syndrome, cryptococcal meningoencephalitis, post-infectious inflammatory response syndrome, primary immunodeficiency, fungal infection

## Abstract

The hyper IgM syndromes are a rare group of primary immunodeficiency. The X-linked Hyper IgM syndrome (HIGM), due to a gene defect in CD40L, is the commonest variant; it is characterized by an increased susceptibility to a narrow spectrum of opportunistic infection. A few cases of HIGM patients with Cryptococcal meningoencephalitis (CM) have been described in the literature. Herein we report the case of a young male diagnosed in infancy with HIGM who developed CM complicated by a post-infectious inflammatory response syndrome (PIIRS), despite regular immunoglobulin replacement therapy and appropriate antimicrobial prophylaxis. The patient was admitted because of a headache and CM was diagnosed through detection of *Cryptococcus neoformans* in the cerebrospinal fluid. Despite the antifungal therapy resulting to negative CSF culture, the patient exhibited persistent headaches and developed diplopia. An analysis of inflammatory cytokines on CSF, as well as the brain MRI, suggested a diagnosis of PIIRS. Therefore, a prolonged corticosteroids therapy was started obtaining a complete resolution of symptoms without any relapse.

## Introduction

Cryptococcal meningoencephalitis (CM) is an opportunistic infection that predominantly affects immunocompromised patients (1). It is amply described in the population affected by HIV/AIDS (2) but also occurs in an increasing number of patients with other forms of natural and iatrogenic immunosuppression such as in primary immunodeficiency and solid organ transplant recipients (3, 4). CD40 ligand (CD40L) deficiency or X-linked Hyper IgM syndrome (HIGM) is a primary immunodeficiency that increases susceptibility to several opportunistic infections. It is caused by a gene defect in the CD40L required for activation of B-lymphocytes and normal production of immunoglobulins as well as T cell activation and differentiation (5). An increased susceptibility to fungal infections has been observed in these patients; *Candida*, *Cryptococcus* and *Histoplasma* are the most commonly implicated (1). Only a few cases of HIGM patients with cryptococcal meningoencephalitis have been described in the literature (6–9) (**Table 1**). Herein we report the case of a young male patient who developed cryptococcal meningoencephalitis complicated by a post-infectious inflammatory response syndrome (PIIRS) while receiving regular antibiotic prophylaxis and subcutaneous immunoglobulin (SCIG) supplementation.

**Table 1:** Clinical and Therapeutic findings in Hyper-IgM Syndrome with CM.

	Malheiro et al.	De Gorgolas et al.	Pacharn et al.	Suzuki et al.	Romani et al.
**Age at diagnosis**	19 yr	27 yr	12 yr	5 yr	22 yr
**Symptoms**	Diplopia/Headache	Fevere/Headeache/vomiting	Fevere/Headeache	Vomiting	Headache
**Wbc (X 10^9^/L/µl) N (%), L (%)**	9.02, N 60, L 21	4.51, N 57	4.050, N 14.3, L 77.6	10.7–44.5, N 64–70	7.47, N 73.5, 16.1
**Antifungal Therapy**	a) liposomal amphotericin and flucytosine b) fluconazole	a) amphotericin B deoxycholate b) fluconazole	a) amphotericin B deoxycholate and fluconazole b) fluconazole	a) amphotericin B deoxycholate	a) liposomal amphotericin and flucytosine b) fluconazole
a) induction phase b) consolidation					
**Steroids**	NA	NA	NA	NA	High dose for 6 days
**Prophylaxis**	Weekly IVIg, TMP-SMZ	IVIg every two weeks, TMP-SMZ	Monthly IVIg, TMP-SMZ	Periodic infusion SGIg	Weekly IVIg, TMP-SMZ, azithromycin
**Outcome**	Complete resolution	Complete resolution	Relapse	Death	Complete resolution

NA, not administrated; Wbc, white blood cell; N, neutrophils; L, lymphocytes; TMP-SMZ, trimethoprim- sulfamethoxazole.

## Case Presentation

A 22 year-old-boy affected by HIGM syndrome (HIGM type 1; OMIM # 308230; CD40L gene) was admitted to a first level hospital because of severe headaches of a two-week duration. He denied fever and was found to exhibit no focal neurological signs. Brain CT scan resulted normal. Suspecting sinusitis, he was initially treated with broad spectrum antibiotics without improvement. Because of worsening headaches, he was transferred to our hospital. Previously, HIGM syndrome had been diagnosed at three years of life during an admission for a *Pneumocystis jiroveci* pneumoniae. The diagnosis was based on a normal lymphocyte T cell count and distribution and absence of CD154 expression in activated CD3+ T lymphocytes. It was then confirmed by Sanger sequencing revealing a c.499G>A; p.G167R mutation in the CD40L gene (NM_000074), a mutation previously described by Asghar Aghamohammadi et al. (10). He was maintained over the years with monthly I.V. immunoglobulin infusions, trimethoprim/sulfamethoxazole (TMP-SMZ), azithromycin and fluconazole prophylaxis without reporting serious infectious except for one episode of *Pseudomonas aeruginosa* otitis. Because of evidence of liver steatosis, the fluconazole was stopped when the patient was twenty years old.

**Figure 1:**
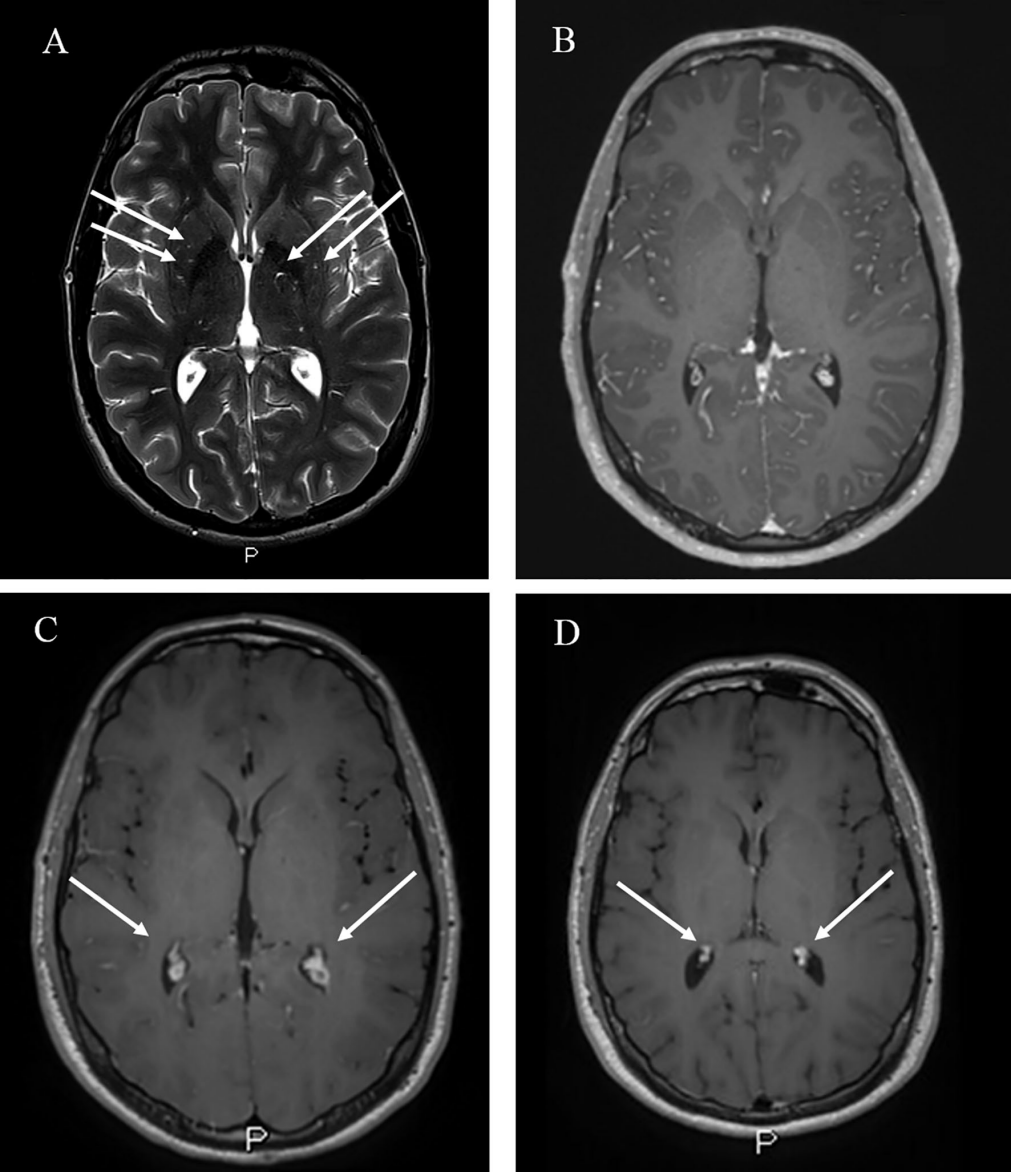
MRI findings of CM in our patients. **(A)** Abnormally enlarged perivascular spaces in the basal ganglia bilaterally (white arrows) in Axial T2-weighted images (WI); **(B)** Choroid plexus (white arrows) at the admission in Axial gadolinium-enhanced T1-WI; **(C)** Bilateral choroid plexitis (white arrows) after ten days of antifungal therapy, in Axial gadolinium-enhanced T1-WI. **(D)** Absence of choroid plexitis after steroids therapy in Axial enhanced T1-WI.

In our hospital the physical examination was normal except for a bilateral mild papillary border elevation. Blood tests showed a white blood cell count of 7,470/µl with 73.5% neutrophils, 16.1% lymphocytes and hemoglobin were 14.3 g/dl, platelet count was 230,000/µl and C-reactive protein was 0.15 mg/dl (normal value <0.50 mg/dl). Brain magnetic resonance imaging (MRI) did not exhibit lesions or evidence suggestive of high intracranial pressure, except for enlarged perivascular spaces in the basal ganglia (**Figure 1**). Therefore, a diagnostic lumbar puncture (LP) was performed. An analysis of cerebrospinal fluid (CSF) showed 78 leucocytes/mm^3^, a glucose level <50% (35 mg/dl) of serum and 84 mg/dl of total protein. Because of a concern for bacterial meningitis, broad-spectrum antibiotic therapy was initiated. Common viruses and bacteria research studies of CSF were negative while cryptococcal antigen of blood and CSF resulted positive with a titer of 1:10 and 1:100, respectively. In addition, CSF cultures were positive for *Cryptococcus* spp, confirmed to be *Cryptococcus neoformans* through polymerase chain reaction (PCR. Blood culture was negative for bacteria and fungi. The patient had no history of contact with birds and he has lived in a city environment since he was a child. Induction therapy with Amphotericin B lipid complex (3 mg/kg q24h) and Flucytosine iv (25 mg/kg q8h) was initiated. Leucopenia and neutropenia were observed after 10 days of therapy, necessitating replacement of flucytosine with fluconazole (1 gr q24h). Despite treatment, the patient exhibited persistent headaches and developed diplopia. Because of suspected high CSF pressures, an external ventricular drain (EVD) was inserted to reduce cerebrospinal pressure resulting in an improvement of headaches; the EVD was removed after a week because of pain in the left lower limb, attributed to the development of lumbar radiculopathy.

After 10 days of combination therapy, an LP was repeated yielding negative CSF cultures and a reduction in cryptococcal antigen titers on CSF (1:10 from 1:100); however, diplopia was still present. Because of diplopia and persistent headaches, in the setting of effective anti-fungal therapy and negative CSF fungal cultures, a post-infectious inflammatory response syndrome (PIIRS) was suspected (11), prompting testing for inflammatory cytokines which were found to be elevated (**Table 2**): CSF studies demonstrated elevated levels of IL6 (1,160 pg/ml) and TNF alpha (241 pg/ml), and continued negative fungal cultures, supportive of PIIRS.

**Table 2:** CSF cytokines measurement before and after corticosteroid salvage therapy (CST).

	Before CST	At two weeks of CST	At 1 months of CST	At 3 months of CST	At 4 months of CST
IL-6 pg/ml	1,160	46	nd	Nd	Nd
IL*-10 pg/ml	Nd	nd	nd	Nd	Nd
TNF alpha pg/ml	241	nd	nd	Nd	Nd

Nd, not detected (<10 pg/ml).

**Figure 2:**
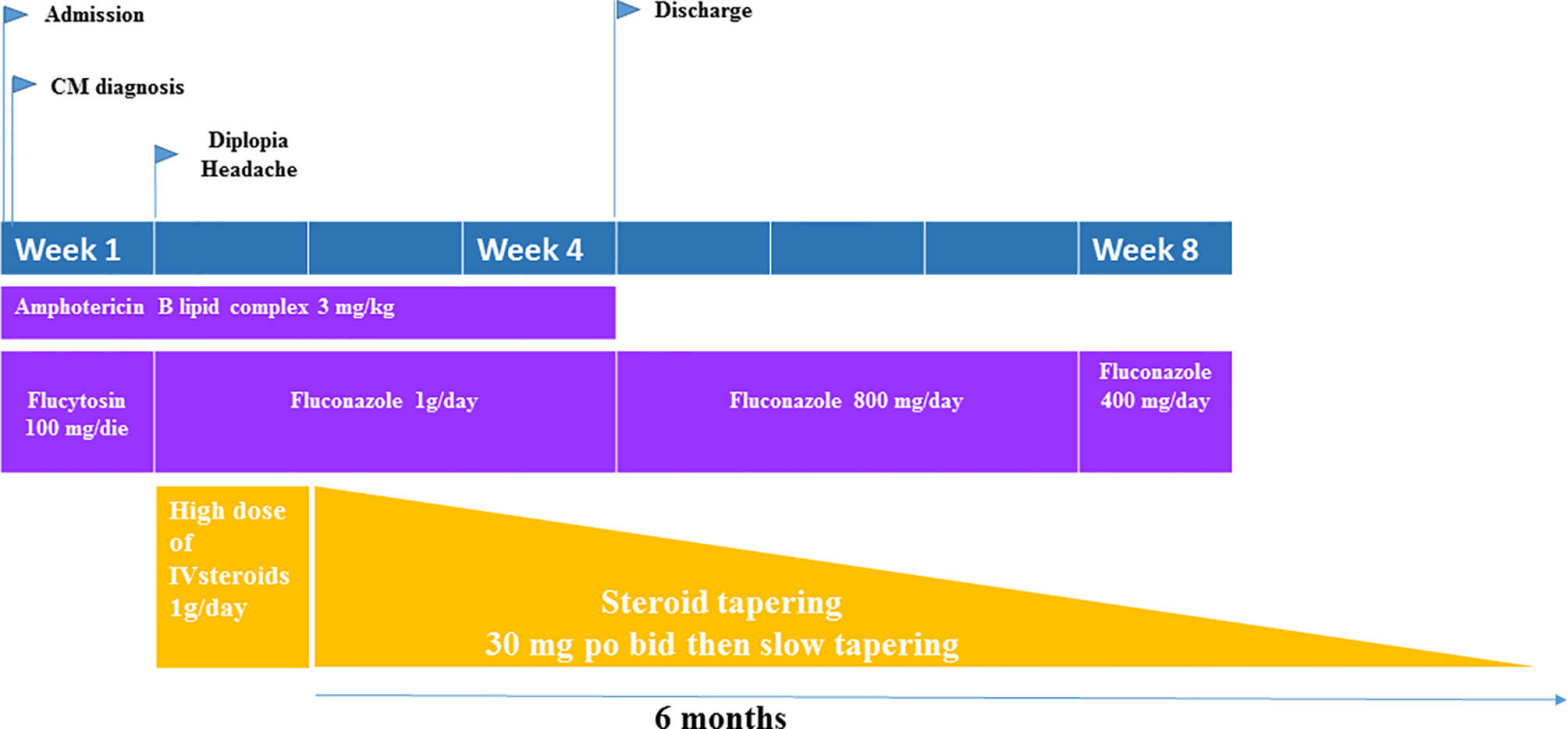
Graphical representation of CM therapy in our patient.

To support the inflammatory hypothesis, as reported by Hammoud et al. (12), brain MRI performed at this stage was conducted which demonstrated hypertrophic choroid plexus (choroid plexitis) (**Figure 1**). Thus, to reduce inflammation, pulse corticosteroid taper salvage therapy was initiated with 6 days of high dose methylprednisolone (1 g) which was slowly tapered over 6 months. Diplopia completely resolved after 6 days of steroids with resolution of papilledema. CSF IL6 levels also declined 25-fold to an undetectable level (<10 pg/ml). Antifungal induction therapy was maintained for 4 weeks and then fluconazole at 800 mg was initiated as consolidation therapy for other 4 weeks. During follow up we repeated the CSF analysis showing normalization of IL6 and TNFα levels and confirming continued microbiological control by negative CSF cultures. The brain MRI performed at the end of steroid therapy also exhibited resolution of previously reported abnormalities (**Figure 1**). The patient was then discharged from the hospital after two weeks from the initiation of steroids therapy, with resolution of diplopia with no other neurological deficits. He continued trimethoprim/sulfamethoxazole prophylaxis (160/800 mg daily, three times a week) plus maintenance fluconazole (800 mg) and continuation of weekly administration of SC immunoglobulin infusions (**Figure 2**). Corticosteroids were tapered over 6 months reducing 5 mg every week. After one year of follow up no neurological sequelae were observed or reported.

## Discussion

CD40 ligand (CD40L) deficiency or X-linked HIGM syndrome is a severe primary immunodeficiency caused by mutations in the CD40L gene and characterized by susceptibility to life-threatening infections (13, 14). CD40–CD40L interaction is an essential signal for B cell proliferation, expression of activation markers, immunoglobulin production, and isotype switching (15) and also plays important roles in the regulation of dendritic cell–T cell (CD4^+^ T and CD8^+^ T) activation and cross-talk (16). The impaired CD40L–CD40 interaction causes susceptibility not only to extracellular bacterial infections but also to opportunistic infections caused by intracellular bacteria, protozoa and fungi (17). Central nervous system (CNS) infections occurred in 6–14% with HIGM syndrome; despite the low incidence they can be fatal or result in severe neurologic sequelae (13, 14). CNS infections such as encephalitis can be caused by *Toxoplasma gondii*, ECHO virus, *Cryptococcus neoformans*, CMV, *Mycobacterium bovis*, *Streptococcus pneumoniae*, *John Cunningham* (JC) virus, and *Pneumococcus* sp (13, 18).

*C. neoformans* is an encapsulated yeast found worldwide that is isolated predominantly from pigeon droppings and soil contaminated with avian excreta (19). It is not unusual for humans to come into contact with it early in life, and a majority of children are likely to have been exposed by the age of 5 (20, 21). However in our case no history of certain contact with birds was reported. Cryptococcal meningoencephalitis (CM) can cause significant morbidity and mortality in previously healthy, non-HIV-infected patients, with all-cause mortality as high as 29% at 1 year (22). Today, a few cases of CM in CD40 deficiency patients are reported in the literature (6–9).

In the experimental study conducted by Petrella et al., it was showed that in mice deficient in CD40/CD40L infected with *C. neoformans*, the antimicrobial activity of macrophages, as well as the magnitude of T and B cell responses were reduced, leading to increased growth of the fungus in brain (23). These elements highlight how the interaction between CD40 and CD40L impairs the development of an efficient immune response against *C. neoformans*.

Recently, research of cryptococcal pathogenesis revealed the contribution of a host immune response to tissue damage (24, 25); indeed, cryptococcal meningitis is considered a classic example of the host damage–response framework (26).

In HIV-related CM, despite microbiological control, a pathological central nervous system inflammatory response occurs after reconstitution of the immune system (IRIS) with antiretroviral therapy (27, 28). Similar to HIV but without immune reconstitution, clinical deterioration following microbiological control in some cases results from a postinfectious inflammatory response syndrome (PIIRS) in HIV negative patients with CM (29–31). This likely is precipitated from released fungal antigens within the intrathecal space that is no longer surrounded by an immune-suppressing polysaccharide capsule. This altered balance results in an excessive host immune response to infection within the space-confines of the skull, causing significant pathology (32), even in the presence of reduced CD40L signaling, as suggested by the present case. During PIIRS an appropriate pro-inflammatory cytokine response including interferon (IFN)-γ and interleukin (IL)-6 stimulates T helper cells to cause immune cells immune mediated host damage but with an alternately activated macrophage subtype (M2) that is less effective at clearing nonviable fungal debris (33, 34). This paradoxical immune response has been studied by Panackal et al. analyzing the soluble and cellular responses to *Cryptococcus* in both blood and CSF in a consecutive cohort of non-HIV, non-transplant individuals with no comorbidities or iatrogenic immunosuppression who developed severe central nervous system disease (s-CNS) (24). Furthermore, they showed a CNS compartimentalized with marked increase in activated T cell and NK cell populations, accompanied by elevated soluble IFN-γ, interferon-generating cytokines IL-18, and interferon-induced chemokines CXCL10 (24), suggesting an investigation of adjunctive T-cell immunosuppressive therapy.

The exact mechanism on how PIIRS developed after cryptococcal meningitis in our patient remains unclear. Susceptibility to human cryptococcal infections is best known to be related to T-cell defects, related to the fungus’ unique encapsulation by a polysaccharide capsule, inert to innate immune recognition by mechanisms including complement and antibody recognition (35). However, after fungicidal therapy by agents such as amphotericin B, rupture of the fungus releases internal proteins and exposes cell wall antigens that provide additional antigenic targets recognizable by the immune-deficient host without a need for immune reconstitution as in HIV-related IRIS (24). Indeed, in a previous report (33), patients with idiopathic CD4 lymphopenia and autoantibodies to granulocyte–monocytic colony stimulating factor (anti-GMCSF), well recognized risk factors for cryptococcal disease (3) developed PIIRS after therapy as in the present case that was also responsive to corticosteroids. Thus, this case extends the spectrum of patients described with PIIRS and the generalizability of this adjuctive therapy in PIIRS.

While the use of corticosteroids (CST) has proven to lack efficacy in patients with HIV-associated CM (36), despite the lack of consensus regarding the management of PIIRS associated to CM, there are data from the literature (30, 33) with evidence of beneficial effect from adjunctive CS therapy (31) to the usual antimicrobial agents.

The use of steroids in CM/PIIRS in HIV-negative patients is reported by Mehta et al. in thirteen patients with persistent neurological symptoms/signs following microbiological control of CM (CSF culture-negative) observing a neurological improvements in five patients (63%) at 1 month of therapy (25). The same result is reported by Anjum et al. in 15 previously healthy patients with CM and PIIRS treated with pulse corticosteroid taper therapy and antifungal therapy (33). In our case we obtained microbiological control after ten days of anti-fungal therapy without resolution of neurological symptoms despite the application of CSF diversion. The introduction of CST improved the clinical response after only 7 days. The tight monitoring of CSF inflammatory cytokines and fungal cultures allowed us to confirm a reduction of inflammatory markers and concurrent microbial control.

In the other cases of CM in HIGM no CST was used (6–9); thus, this report is the first description of the use of CST in CM within this patient population, extending the safety and efficacy of corticosteroids in PIIRS within this immune-suppressed patient population.

The addition of CST in these patients requires careful attention as CST suppresses innate and acquired immune responses (37) and long-term use may thus increase the risk of recurrence of CM or co-infection in a population already compromised. In this context, although there is no standard guideline for secondary cryptococcal prophylaxis in primary immune-deficiency patients, we decided to continue the antifungal secondary prophylaxis.

Hammoud et al. (12) compared the brain MRI between CM HIV positive (+) and CM HIV negative (–) patients describing as addition findings in the HIV—patients, choroid plexitis and ependymitis as well as pachymeningeal enhancement and ischemic infarcts. Furthermore, they support the hypothesis that these findings reflect a post-infectious inflammatory response syndrome (PIIRS) rather than microbiological failure since the majority of the patients had negative fungal CSF cultures. Indeed, in our case the evidence of choroid plexitis was reported when the CSF was negative for *C. neoformans* and inflammatory cytokines were elevated.

## Conclusion

In conclusion, our case emphasized that *C. neoformans* should be considered among etiological agents in patients with X-linked HIGM syndrome presenting with symptoms and signs of central nervous system involvement. CM might be complicated by PIIRS, therefore it should be suspected when there is no clinical improvement despite the antimicrobial control. The analysis of CSF soluble biomarker should be performed in order to consider the use of CST as salvage therapy.

Further investigations are needed to identify treatment strategies for non-HIV patients with CM in order to reduce damage caused by both the microbe and the pathological immune response.

## Data Availability Statement

The original contributions presented in the study are included in the article/**Supplementary Material**. Further inquiries can be directed to the corresponding author.

## Ethics Statement

Written informed consent was obtained from the individual(s) for the publication of any potentially identifiable images or data included in this article.

## Author Contributions

LR and AF wrote the manuscript. GM, SC and RC performed the experiments and analyzed the data. PW contributed to the data interpretation and writing. LF-T reviewed the radiological findings. PR, CC, ML, LR, and AF followed up on the patient.RC performed and analyzed CSF cytokines. All authors contributed to the article and approved the submitted version.

## Funding

This work was partially supported by the NIH Intramural Research Program and by Bambino Gesù Children’s Hospital Research Program (RIC-2020 – PI AF).

## Conflict of Interest

The authors declare that the research was conducted in the absence of any commercial or financial relationships that could be construed as a potential conflict of interest.
