# Supplementary material for: Cryptococcal Meningitis and Post-Infectious Inflammatory Response Syndrome in a Patient With X-Linked Hyper IgM Syndrome: A Case Report and Review of the Literature
Source: Front Immunol. 2021 Jul 15;12:708837. doi: 10.3389/fimmu.2021.708837 (PMC8320724; doi:10.3389/fimmu.2021.708837)
Supplement: Supplementary file 1 [file DataSheet_1.pdf]

Supplementary material

Figure 1. CD154 expression on resting and activated PBMC gated on CD3+ T cells

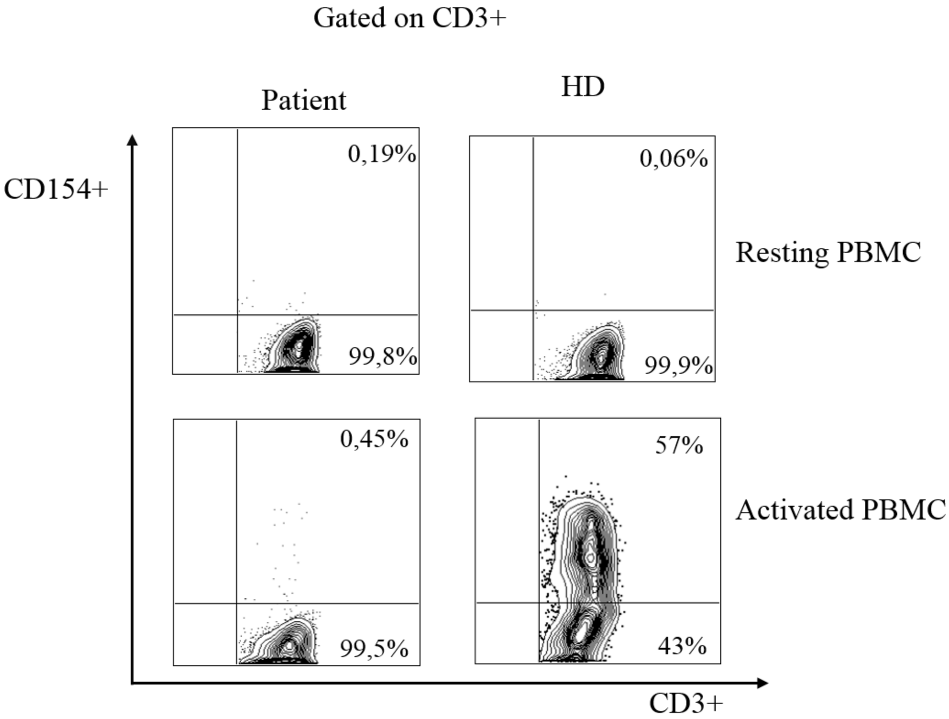

Table 1. CSF features at three times points: at the admission, after high dose of steroid treatment and during the steroid tapering

CSF	Admission	After high dose of steroid treatment	During steroid tapering
**Glucose** mg/dl	35	64	45
**Total protein** mg/dl	84	61	45
**WBC** mmc	78	18	3
**Cryptococcal antigen titre**	1:100	1:1	Positive, no titre

Table 2| Patient's immunological investigations at 20 years old

**COMPLETE BLOOD COUNT**	Patient value	Normal age-matched value
Complete blood count		
White blood cell (10^3 /µl)	4.15	4.00–11.00
Red blood cells (10^6/µl)	5.42	4.40–6.00
Neutrophils (10^3 /µl)	1.10	1.60–7.70
Lymphocytes (10^3 /µl)	2.47	0.80–4.73
Platelets (10^3 /µl)	204	150–450
Hemoglobin (g/dl)	14.9	13–17.50
**LYMPHOCYTES SUBSETS**	Patient value	Normal age-matched value
Absolute CD3+ (/µl)	2129	(2100-6200)
Absolute CD4+ (/µl)	617	(608–1217)
Absolute CD8+(/µl)	1259	(228–577)
Absolute CD19+ cells/mL(/µl)	222	(119–578)
Absolute CD16+CD56+ (/µl)	83	(720.2600)
**% on total lymphocytes (absolute value)**		
CD3+ CD45+	**86,2**	71 (62-81) §
CD19+ CD45+	9,0	12 (6-21) §
NK (CD3- CD16+CD56+ CD45+)	3,4	16 (6-23) §
CD3+ CD4+	**25,5**	42 (31-53) §
CD3+ CD8+	**51**	26 (19-30) §
**% on CD3+CD4+ T cells**		
CD3+CD4+CD27+CD45RA+Naïve CD4+	63,7	62.0 (49.4–71.9) °
CD3+CD4+CD27+CD45RA- Central Memory	29,8	33.8 (24.3–42.7) °
CD3+CD4+CD27-CD45RA- Effector Memory	6,0	3,2 (2,1-7,4) °
CD3+CD4+CD31+CD45RA+ Effector Memory CD45RA+	0,44	0,3 (0,1-7,4) °
CD3+CD4+CD31+CD45RA+ Recent Thymic Emigrants	59,2	42.8 (32.7–60.9) °
CD4+CD45RO+CXCR5+ Peripheral T helper follicular	**0,7**	5,6 (3,1-9,1) #
% on CD3+CD8+ T cells		
CD3+CD8+CCR7+CD45RA+ Naïve CD8+	12,4	43 (18-61) §
CD3+CD8+CCR7+CD45RA- Central Memory	0,63	6 (3-12) §
CD3+CD8+CCR7-CD45RA-Effector Memory	68,4	39(25-58) §
CD3+CD8+CCR7-CD45RA+ Effector Memory CD45RA+	18,6	12 (5-20) §
**% on CD19+ B cells**		
CD27+IgD+IgM+ unswitched memory	**1,1**	6.4 (2.6–13.4) ^
CD27+IgD-IgM- Switched memory	**0,45**	9.1 (4.0–21.2) ^
CD27-IgD+IgM+ naïve B cells	**96,6**	79.6 (61.6–87.4) ^
CD27-IgD-IgM- double Negative B cells	**1,93**	4.9 (1.4–13.0) ^
MEMORY CD19+CD27+ Total Memory B cells	**1,4**	16.0 (7.0–29.0) ^

LYMPHOCYTE STIMULATION TO MITOGENS (cpm)	Patient value	Normal age-matched value
Phytohemagglutinin	>35.000	>35.000
**SERUM IMMUNOGLOBULIN LEVELS**		
IgG (mg/dL)	**783**∞	700-1600
IgA (mg/dL)	**247**	70-400
IgM (mg/dL)	**126**	40-230

^ Duchamp, M., et al. (2014), B-cell subpopulations in children: National reference values. Immunity, Inflammation and Disease, 2: 131-140.

° van Gent R, et al. Refined characterization and reference values of the pediatric T- and B-cell compartments. Clin Immunol. 2009 Oct;133(1):95-107.

§ Garcia-Prat M, et al. Extended immunophenotyping reference values in a healthy pediatric population. Cytometry B Clin Cytom. 2018 Oct 17.

∞ On IVIG

Internal laboratory reference values
